# Comment on “Emergence of Hepatitis B Virus Genotype F in Aligarh Region of North India”

**DOI:** 10.1155/2014/637439

**Published:** 2014-03-17

**Authors:** Sibnarayan Datta

**Affiliations:** Defence Research Laboratory, Defence R & D Organization (DRDO), Ministry of Defence Government of India, Tezpur, Assam 784 001, India

I read with interest the recent research article by Sami and colleagues, entitled “Emergence of Hepatitis B Virus Genotype F in Aligarh Region of North India” published in the Advances in Virology. In this paper the authors report the emergence of hepatitis B virus (HBV) genotype F in North India.

The paper is of significant importance from the molecular epidemiological perspective, since circulation of HBV genotypes A, C, and D has already been established through studies by our group and other groups working in this field. In continuation, emergence of HBV genotype F also signifies the changing molecular epidemiology of HBV in the Indian subcontinent.

As evident from the title, it appears that the primary aim of the authors was to report the emergence of HBV genotype F in India. The authors report that HBV genotype D was the most prevalent genotype in their study, which corroborates with published data from the same region. In contrast, authors report that genotype F was detectable in only 5 patients. However, the description of genotyping scheme seems a bit confusing, since a number of isolates were not type-able by the used methods. Interestingly, 5 HBV genotype F isolates were identifiable by Naito's method, but only 1 of those 5 could be confirmed by the method described by Kirschberg et al. Moreover, despite the emergence of HBV genotype F being at the central theme of the research paper, the authors did not attempt to sequence even a singular amplicon that was amplifiable by either of the HBV genotype F specific PCR. In contrast, they sequenced 10 HBV genotype D amplicons and subsequently subjected those sequences to phylogenetic analysis. Confirmation of HBV genotype D through sequencing and phylogenetic analysis does not provide any new data, since the prevalence of this genotype in Northern India has already been established by the findings of other research groups. Nevertheless, the authors did neither mention if the sequence data generated in this study were submitted to the GenBank nor they provided the GenBank accession numbers.

Lastly, HBV genotype F reference sequence used in the phylogenetic analysis (GenBank accession number AY090461) was isolated from El Salvador, as evident from the GenBank records, not from Sweden, as mentioned by the authors. Moreover, reference sequences AY373429 belong to HBV genotype A, not Genotype D, as mentioned by the authors. The authors are expected to be more careful towards selection of reference sequences, their GenBank annotations, and phylogenetic analysis.

Considering the above facts, the findings and the conclusions of the abovementioned research paper remain dubious.

